# Algae as New Kids in the Beneficial Plant Microbiome

**DOI:** 10.3389/fpls.2021.599742

**Published:** 2021-02-04

**Authors:** Sang-Moo Lee, Choong-Min Ryu

**Affiliations:** ^1^Molecular Phytobacteriology Laboratory, Infectious Disease Research Center, KRIBB, Daejeon, South Korea; ^2^Department of Biosystems and Bioengineering, KRIBB School of Biotechnology, University of Science and Technology, Daejeon, South Korea; ^3^Department of Applied Bioscience, Dong-A University, Busan, South Korea

**Keywords:** microalgae, microbiome, *Chlorella*, cyanobacteria, plant immunity, plant growth promotion (PGP), biological control

## Abstract

Previously, algae were recognized as small prokaryotic and eukaryotic organisms found only in aquatic habitats. However, according to a recent paradigm shift, algae are considered ubiquitous organisms, occurring in plant tissues as well as in soil. Accumulating evidence suggests that algae represent a member of the plant microbiome. New results indicate that plants respond to algae and activate related downstream signaling pathways. Application of algae has beneficial effects on plant health, such as plant growth promotion and disease control. Although accumulating evidence suggests that secreted compounds and cell wall components of algae induce physiological and structural changes in plants that protect against biotic and abiotic stresses, knowledge of the underlying mechanisms and algal determinants is limited. In this review, we discuss recent studies on this topic, and highlight the bioprotectant and biostimulant roles of algae as a new member of the plant beneficial microbiome for crop improvement.

## Introduction

Algae is a group of ancient photosynthetic organisms ranging from prokaryotic cyanobacteria to eukaryotic microalgae (Parker et al., 2008). Generally, algae are classified mainly depending on their color, shape, and life cycle (Blaby-Haas and Merchant, 2019). Out of more than 800,000 species of algae that exist in nature, only 5,000 have been characterized to date. Out of 5,000 species, only small number of the algae species have been selected to determine their potential applications in plant growth under defined growth conditions. Algae are broadly classified as micro- and macroalgae based on size. Macroalgae indicates large aquatic photosynthetic plants that can be seen without the aid of a microscope and can generally be divided into three groups: Green (Chlorophyta), Red (Rhodophyta), and Brown-Kelps (Phaeophyta—related to Chromista). Microalgae comprise representative genera, including *Arthrospira*, *Chlorella*, *Dunaliella, Nostoc*, and *Aphanizomenon* (Elster, 2002). Prokaryotic microalgae, namely, cyanobacteria, play a critical role in the natural ecosystem, particularly in plant–microbe interactions. However, the idea that algae are a member of the plant-associated microbial community has long been debated (Berg et al., 2020).

## Definition and Membership of the Microbiome

It is important to understand the definition of the microbiome before discussing algae as a new member of the plant microbiome, since microbe and microbiome are distinct terminologies. Most scientists follow the definition of microbiome first provided by Whipps et al. (1988), according to which a microbiome “may be defined as a characteristic microbial community occupying a reasonably well defined habitat which has distinct physio-chemical properties. This term not only refers to the microorganisms involved but also encompasses their theater of activity” (Whipps et al., 1988). However, the definition of microbiome has been revised several times in the last 20+ years to meet the technological and conceptual advances. “The microbiome is defined as a characteristic microbial community occupying a reasonable well-defined habitat which has distinct physio-chemical properties. The microbiome not only refers to the microorganisms involved but also encompass their theater of activity, which results in the formation of specific ecological niches. The microbiome, which forms a dynamic and interactive micro-ecosystem prone to change in time and scale, is integrated in macro-ecosystems including eukaryotic hosts, and here crucial for their functioning and health” (Berg et al., 2020).

Many microbiologists less considered algae and protists as members of the plant-associated microbiome (Longford et al., 2019; Wilpiszeski et al., 2019). However, most microbiologists agree that algae, except some macroalgae, are microorganisms based on their size and characteristics. In this review, we discuss only microalgae species, including both prokaryotic and eukaryotic organisms. The ecological niche of algae had also been debated. Here, we focus on algae as a member of the microbiome and their beneficial effects on plant fitness. To meet the minimum conceptual role, algae must exist on or around the plant surface and inside plant tissues.

## Algae as Members of the Soil Microbiome

Because fresh and seawater were previously recognized as the habitat of algae, most microbiologists did not consider that algae could thrive in soil or on plant surfaces. However, more than 30 years ago, scientists investigated the distribution of algae in soil (Davey, 1989, 1991; Davey and Clarke, 1991). Early studies were conducted to identify cryptogrammic flora on the Antarctic fellfield soil based on their chlorophyll contents and microscopic observations. These studies revealed that Oscillatoriaceae was the dominant family in the soil, up to a depth of 8 cm below the soil surface (Davey and Clarke, 1991). Limitations of the classification on algal species based on conventional microbiological approaches, including isolation and *in vitro* culture on artificial media, led to the development of molecular techniques, including PCR-based 18S rDNA sequencing of the algae community in the soil (Bérard et al., 2005; Bradley et al., 2016; Khaw et al., 2020). In areas with harsh climatic conditions, such as semi-arid steppes, warm deserts, and polar regions, the algal community forms a biological soil crust along with other microorganisms to protect against abiotic and biotic stresses (Zhang et al., 2011; Pushkareva et al., 2016; Krug et al., 2020). Algae were also identified as active microbes in agricultural fields by 18S rDNA sequencing (Bérard et al., 2005). For instance, four classes of algae were identified in soil samples collected from a vegetable field (depth: 0–15 cm) in Nigeria: Chlorophyceae, Cyanophyceae, Bacillariophyceae, and Euglenophyceae (Adesalu and Olugbemi, 2015). Collectively, these studies suggest that algae are distributed across diverse environments, ranging from polar areas to agricultural fields. However, the interaction between land plants and algae has not been studied intensively. To utilize algae as plant health-promoting factors, it is important to understand the ecological niche of algae.

## Ecological Niche

Previously, freshwater and seawater were considered as the ecological niches of algae, as described above. Considering algae as a member of the plant microbiome (phytobiome) has been debated because algae could not be isolated from the rhizosphere, phyllosphere, or endosphere (Gantar and Elhai, 1999; Gantar, 2000; Treves et al., 2016; Zhu et al., 2018). Moreover, the role of algae in plant fitness has not been evaluated extensively by biochemical and molecular analyses. Only recent studies demonstrate that algae are a member of the phytobiome. For instance, *Chlorella* species are found in the soil and on the plant leaf surface (Liu and Chen, 2016; Treves et al., 2016; Zhu et al., 2018), and cyanobacteria, such as *Nostoc* and *Anabaena* spp., were identified on the plant root surface (Gantar et al., 1991, 1995; Spiller et al., 1993; Gantar and Elhai, 1999; Gantar, 2000). However, recent microbiome analysis using the DNA sequence-based metagenome technology revealed that microalgae, including eukaryotic and prokaryotic (cyanobacteria) species, must be considered as members of the microbiome (Mendes et al., 2013; Xu et al., 2018). Microalgae have also been identified in the soil and in plant tissues (Leach et al., 2017). Previous studies on plant–algae interactions did not demonstrate the beneficial effects of algae on plant growth and defense. In this review, we focus on algae as a member of the beneficial microbiome and on their beneficial effects on plant health. Since the concept of ‘beneficial microbiome’ has not been defined clearly (Berg et al., 2020), beneficial algae could be categorized as having direct and indirect beneficial effects on plant, similarly to other beneficial microbes (e.g., PGPR). The bacterial and fungal inoculants on seeds, seedlings, and propagating plant materials secrete growth-enhancing compounds directly, which mimic plant hormones and promote increased plant growth and yield (Lugtenberg and Kamilova, 2009). The inoculants also promote plant growth by inhibiting pathogenic and deleterious plant-associated microbes and by activating plant innate immunity against plant pathogens; the latter represents an indirect effect of beneficial bacteria and fungi on plants. Another indirect effect of such inoculants is modulation of the microbiome, referred to as microbiome engineering (Dessaux et al., 2016). The direct and indirect effects of bacteria and fungi on plants are well known, but those of algae are a new emerging concept. Here, we summarize the beneficial effects of algae on crop plants in the greenhouse and field.

## Plant Root Colonization and Para-Nodule Formation

**FIGURE 1:**
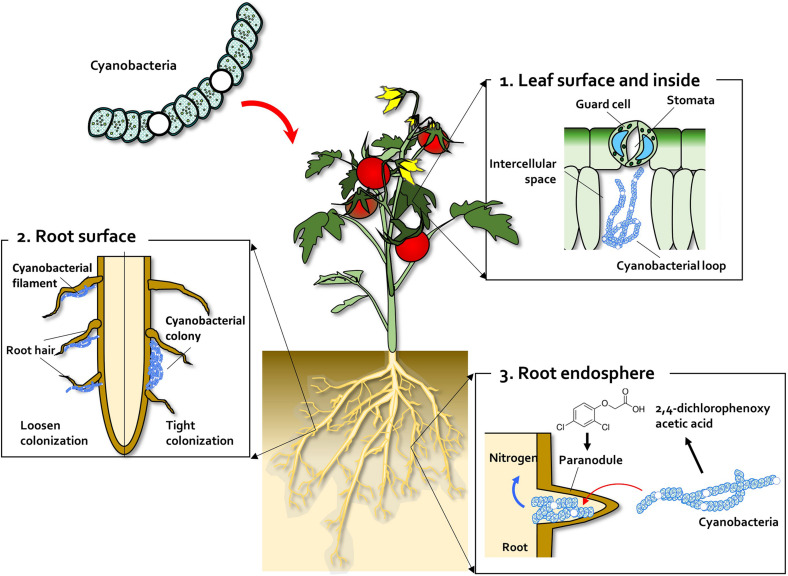
Leaf and root colonization by cyanobacteria. **(1)** Cyanobacteria enter the leaf tissue through the stomata and colonize the intercellular space, forming a cyanobacterial loop. **(2)** On the root surface, cyanobacteria exhibit two types of colonization pattern; in the root hair, filaments of *Anabaena* and *Nostoc* species form loose colonies, and in the restricted zone on the root surface, specific *Nostoc* species form cyanobacterial colonies. **(3)** Co-inoculation with 2,4-dichlorophenoxy acetic acid (2,4-D) (synthetic auxin) and *Nostoc* spp. increases *para*-nodule formation and nitrogen fixation. A large number of *Nostoc* spp. isolates colonize the root endosphere and form *para*-nodules to fix nitrogen.

Many species, ranging from moss to angiosperms, exhibit symbiotic interactions with algae (Meeks and Elhai, 2002; Santi et al., 2013). To interact with plants, algae must colonize the plant surface and cells within plant tissues, similar to other microbial organisms involved in symbiotic and mutualistic interactions with plants (Figure 1). Most examples of plant–algae interactions involve prokaryotic algae, i.e., cyanobacteria (Gantar and Elhai, 1999; Gantar, 2000; Treves et al., 2016; Zhu et al., 2018). Cyanobacteria can enter the plant through the stomata and colonize the intercellular space, forming loops and intracellular coils (Krings et al., 2009) (Figure 1). *Anabaena* spp. colonize the roots of wheat and cotton plants (Karthikeyan et al., 2009; Babu et al., 2015; Bidyarani et al., 2015) (Figure 1). *Calothrix* sp. was also found on the root system of wheat (Babu et al., 2015; Bidyarani et al., 2015). Beyond colonization of the root surface, *Tolypothvix* sp. and *Leptolyghya* sp. were detected in the intercellular space in Cycads plants (Cuddy et al., 2012) (Figure 1). Thus, the algae–plant interactions represent another example of a symbiotic relationship between the two organisms. A good example of this relationship is colonization of monocots, such as wheat and rice, by *Nostoc* spp. (Gantar et al., 1991; Ahmed et al., 2010; Hussain et al., 2013, 2015). Gantar et al. (1991) isolated diverse heterocystous nitrogen-fixing cyanobacteria, including *Nostoc*, *Anabaena*, and *Cylindrospermum*, from plant root and soil. Assessment of wheat seedling roots revealed two types of association patterns: loose colonization of root hair by *Anabaena* and tight colonization of the root surface within a restricted zone by *Nostoc* (Gantar et al., 1991) (Figure 1).

In addition to the free-living lifestyle on the plant root surface, *Nostoc* species also exist as endosymbionts in the flowering land plant (angiosperm) *Gunnera* (Silverster and Smith, 1969; Silvester, 1976; Lindblad et al., 1990). Cyanobacteria also form symbiotic relationships with other plant species, including bryophytes (e.g., *Anthoceros*), gymnosperms (e.g., Cycads), and water fern (*Azolla*) (Braun-Howland and Nierzwicki-Bauer, 1990; Lindblad et al., 1990; Meeks and Rai, 1990). Among these four multicellular plants, *Gunnera* L. develops well-organized, unique organs named glands through symbiosis with *Nostoc* (Bergman et al., 1992). Intriguingly, the glands of *Gunnera* are morphologically similar to crown galls formed by *Agrobacterium tumefaciens*. The detailed mechanisms by which the following processes happen, have been elucidated as gland morphology, cell penetration, intracellular colonization, hormogonium formation, gland development, and host specificity. During symbiosis, cyanobacteria influence nitrogen fixation and release, heterocyst development, and consistence of symbiosis. Glands formed by *Nostoc* on the stem of *Gunnera* plants are similar to nodules formed by *Rhizobium* spp. and crown gall formed by *A. tumefaciens* (Rasmussen et al., 1996). The *Nostoc* genome does not contain homologs of the two *Agrobacterium* genes required for T-DNA transfer-induced crown gall formation in plants, indicating that the mechanism of gland formation is distinct from that of gall formation. By contrast, the genome of *Nostoc* harbors homologs of the *Rhizobium* nod-box genes including *nodEF*, *nodMN*, and *enoY*. However, the induction of other critical nod genes, including *nodABC*, *nodD1*, and *nodD2*, and nod protein, could not be detected in *Nostoc* when treated with acidic mucilage secreted by stem glands (Rasmussen et al., 1996). These data indicate that *Rhizobium*–legume symbiosis is distinct from *Gunnera–Nostoc* symbiosis.

Scientists have attempted to form nodule-like structures and to functionally fix nitrogen in non-legume plants. Tchan and Kennedy (1989) succeeded in developing nodule-like structures, named para-nodules, using 2,4-dichlorophenoxy acetic acid (2,4-D), a synthetic compound that mimics auxin, but they failed to fix nitrogen using nitrogenase-containing bacteria. Inoculation of *Nostoc* sp. strain 2S9B into the 2,4-D led para-nodule increased the acetylene reduction capacity by more than threefold compared with that of the untreated control (Gantar and Elhai, 1999) (Figure 1). In the absence of supplemental nitrogen, wheat shoot growth could be increased by co-inoculation with 2,4-D and *Nostoc* sp. strain 2S9B (Gantar and Elhai, 1999; Gantar, 2000). Similarly, para-nodule formation and nitrogen fixation could also be induced in rice seedlings by treatment with 2,4-D and *Nostoc* spp. (Nilsson et al., 2002). Two possibilities could explain why para-nodules do not occur naturally in land plants such as wheat and rice: (i) below-threshold levels of auxin, and (ii) lack of *Nostoc* spp. colonization on the wheat and rice tissues as the ecological niche (Figure 1). This can be used to identify specific *Nostoc* spp. that elicit para-nodule formation in land plants in near future. Intriguingly, unlike prokaryotic algae, it is not reported that eukaryotic algae colonize on plant tissues.

## Algae as a New Member of the Beneficial Plant Microbiome

### Biological Control of Plant Pathogens

**FIGURE 2:**
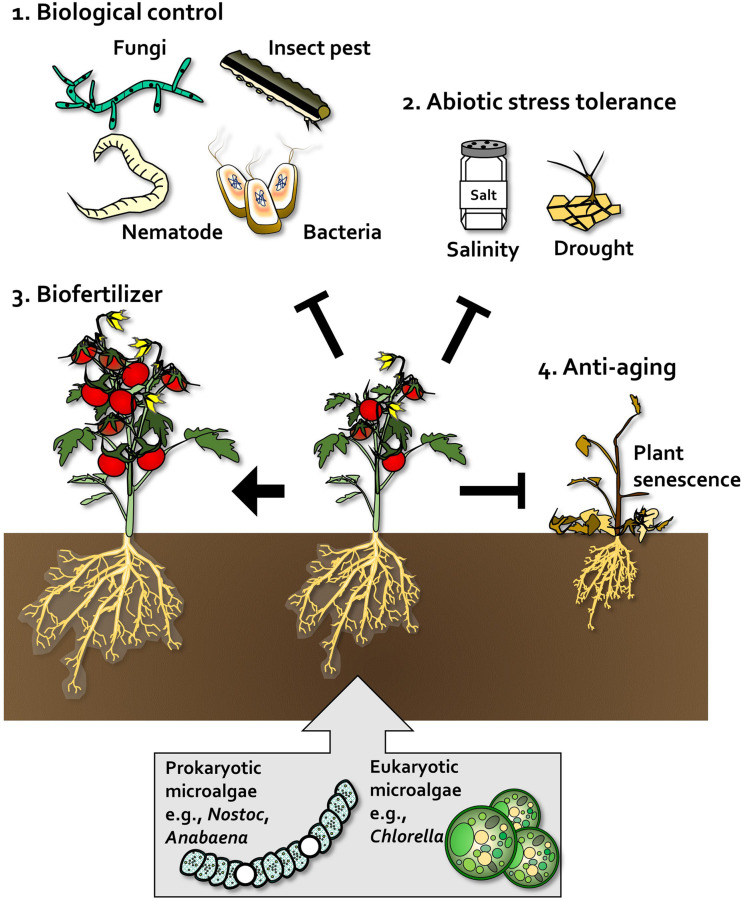
Beneficial effects of algae on plants. In plants, prokaryotic microalgae such as *Nostoc* and *Anabaena*, and eukaryotic microalgae such as *Chlorella*, act as biological control agents **(1)**, abiotic stress tolerance enhancers **(2)**, biofertilizers that promote plant growth and crop yield **(3)**, and anti-aging agents that delay senescence and enhance plant robustness **(4)**.

Algal species have been used intensively for biological control of fungal pathogens (Figure 2 and Table 1). In tomato and cotton, root-drench application of prokaryotic *Anabaena variabilis*, *Anabaena torulosa*, *Anabaena laxa*, and *Calothrix* sp. reduced damping-off symptoms caused by *Pythium debaryanum*, *Fusarium oxysporum*, *F. moniliforme*, and *Rhizoctonia solani* (Prasanna et al., 2008, 2013; Chaudhary et al., 2012) (Figure 2 and Table 1). Additionally, the eukaryotic *Chlorella fusca* protects host plant against pathogenic fungi *Colletotrichum orbiculare* and *Botrytis squamosa* in cucumber and Chinese chive (Lee et al., 2016, 2017; Kim et al., 2018a). The cell extract or filtered supernatant of cyanobacteria and *Chlorella* species also exhibits biological control activity against *F. oxysporum*, *P. aphanidermatum*, and *Sclerotinia sclerotiorum* in tomato, pepper, and brinjal (Biondi et al., 2004; Kim and Kim, 2008; Manjunath et al., 2010). Algae can suppress fungal disease via two putative mechanisms. First mechanism involves inhibition of fungal pathogen growth (Figure 2 and Table 1). For example, cyanobacteria *Anabaena* and *Calothrix* species showed antagonistic activity against *Fusarium* spp., *Pythium* spp., and *Rhizotoctonia* spp. *in vitro* (Chaudhary et al., 2012; Prasanna et al., 2013, 2016), and eukaryotic *C. fusca* also inhibited the growth of *C. orbiculare* hyphae *in vitro* and suppressed the formation of appressorium on cucumber leaves (Lee et al., 2016, 2017). The second mechanism involves activation of plant immune responses. *C. fusca* treatment showed antagonistic activity against *C. orbiculare* as well as the induction of defense-related structural modifications such as cell wall thickness, vesicle accumulation, and sheath formation, in cucumber leaves (Kim et al., 2018b).

**TABLE 1:** Biological control of plant pathogens and insects using algal species.

Group	Algae species/consortia	Pathogen/insect type	Pathogen/insect name	Host plant	Treatment method	Product applied	References
Prokaryotic cyanobacteria	*Anabaena variabilis* RPAN59, *Anabaena laxa* RPAN8	Fungal pathogen	*Pythium debaryanum, Fusarium oxysporum* f. sp. *lycopersici, Fusarium moniliforme*, and *Rhizoctonia solani*	Tomato	Soil application	Cell culture and filtered supernatant	Chaudhary et al., 2012; Prasanna et al., 2013
	*Anabaena torulosa, A. laxa, Calothrix* sp.	Fungal pathogen	*R. solani*	Cotton	Soil application	Cell culture	Prasanna et al., 2016
	*Nostoc commune* FA-103	Fungal pathogen	*F. oxysporum* f. sp. *lycopersici*	Tomato	Seed coating	Cell extract	Kim and Kim, 2008
	*Calothrix elenkenii*	Fungal pathogen	*Pythium aphanidermatum*	Tomato, chili, and brinjal	Seed soaking	Filtered supernatant	Manjunath et al., 2010
	*Nostoc* strain ATCC 53789	Fungal pathogen	*Sclerotinia sclerotiorum*	Tomato	Seed soaking	Cell biomass and methanolic extract	Biondi et al., 2004
	*Microcoleus vaginatus*	Pathogenic nematode	*Meloidogyne arenaria*	Tomato	Root dipping	Filtered supernatant	Khan et al., 2005
	*Oscillatoria chlorina*	Pathogenic nematode	*M. arenaria*	Tomato	Soil application	Dried cell suspension	Khan et al., 2007
	*Aphanocapsa albida, Anabaena oryzae, Nostoc muscorum*, and *Calothrix marchica*	Pathogenic nematode	*Meloidogyne incognita*	Tomato	Soil application	Aqueous extract	Hamouda and El-Ansary, 2013
	*Scytonema* MKU 106	Chewing insect	*Helicoverpa armigera, Heliothis* larvae, and *Sylepta derogata*	Cotton	Foliar application	Peptide extract	Sathiyamoorthy and Shanmugasundaram, 1996
Eukaryotic green algae	*Chlorella vulgaris*	Pathogenic nematode	*M. arenaria* and *Xiphinema indexin*	Tomato, grape	Soil application	Dried cell extract	Choleva et al., 2005; Bileva, 2013
	*Chlorella fusca*	Fungal pathogen	*Colletotrichum orbiculare*	Cucumber	Foliar application	Cell culture	Lee et al., 2016, 2017; Kim et al., 2018a
	*C. fusca*	Fungal pathogen	*Botrytis squamosa*	Chinese chives	Foliar or soil application	Cell culture	Kim et al., 2018b
	*C. fusca*	Bacterial pathogen	*Pseudomonas syringae* pv. *tomato*	Arabidopsis	Foliar application	Cell culture/cell-free supernatant	Lee et al., 2020a
Cyanobacteria–bacteria consortia	*Anabaena oscillarioides* and *Bacillus subtilis*	Fungal pathogen	*F. oxysporum, P. debaryanum, P. aphanidermatum*, and *R. solani*	Tomato	Soil application	Cell culture	Dukare et al., 2011

Microalgae species have also been used to control pathogenic nematodes and insect pests (Sathiyamoorthy and Shanmugasundaram, 1996; Choleva et al., 2005; Khan et al., 2005, 2007; Bileva, 2013; Hamouda and El-Ansary, 2013) (Figure 2 and Table 1). Root treatment of tomato with cyanobacteria such as *Microcoleus vaginatus*, *Oscillatoria chlorine*, *Aphanocapsa albida*, *Anabaena oryzae*, *Nostoc muscorum*, and *Calothrix marchica* reduced gall formation caused by *Meloidogyne arenaria* and *M. incognita* (Khan et al., 2005, 2007; Hamouda and El-Ansary, 2017). Soil-drench application of *Chlorella vulgaris* extract (1 g per pot) reduced infestation of grapevine roots by *Xiphinema index* by 2–3-fold compared with the untreated control (Choleva et al., 2005, 2007; Bileva, 2013). Foliar application of 0.01–0.1% peptides extracted from the cyanobacterium *Scytonema* MKU 106 reduced the feeding frequency of a chewing insect, *Sylepta derogata*, in cotton plants (Sathiyamoorthy and Shanmugasundaram, 1996). The algae species can protect host plant against pathogenic nematode and insect pests by nematocidal or repellent activity rather than as plant immune activation (Sathiyamoorthy and Shanmugasundaram, 1996; Choleva et al., 2007).

Compared with fungal pathogens and insect pests, biological control of bacterial pathogens using algae has remained largely unknown until 2020, when we reported for the first time the biological control of the bacterial pathogen *Pseudomonas syringae* pv. *tomato* (*Pto*) by *C. fusca* in the model plant, *Arabidopsis thaliana* (Lee et al., 2020a, Figure 2 and Table 1). Foliar application of *C. fusca* culture (10^7^ cells/ml) reduced the population of *Pto* in *Arabidopsis* leaves by 10-fold. Further investigation revealed that *C. fusca* and its determinant D-lactic acid prime plant innate immunity against *Pto* (Lee et al., 2020a). To the best of our knowledge, there have been no reports on the biocontrol activity of algae against phytopathogenic viruses. Therefore, testing the potential application of algae against plant viruses is important.

### Induced Tolerance Against Abiotic Stresses

The application of algae-derived substances could also increase tolerance against abiotic stresses (Figure 2 and Table 2). In rice, extracellular products of the cyanobacterium *Scytonema hofmanni* nullified the effects of salt stress (<5 g/ml NaCl) on dry weight and length of shoot (Rodríguez et al., 2006). Under high salt stress, tomato plants treated with 1% *Dunaliella salina* hydrolyzate via spray application showed higher shoot dry weight, root dry weight, and chlorophyll a and b content than untreated plants (Arroussi et al., 2018), and soil treatment with water-soluble extracts of *Chlorella ellipsoida* and *Spirulina maxima* increased the total protein content of wheat grain by 1.4-fold compared with the control (Abd El-Baky et al., 2010). Moreover, treatment of fava bean plants with *C. vulgaris* culture induced drought tolerance (Li et al., 2014). Abiotic stress tolerance triggered by microalgae treatment is mostly linked to production of reactive oxygen species (ROS) and antioxidant activity in plants (Li et al., 2014; Arroussi et al., 2018). In tomato and bean, foliar application of *D. salina* extracts and *C. vulgaris* activated antioxidant enzymes such as peroxidase (POD), superoxide dismutase (SOD), catalase (CAT), and ascorbate peroxidase (APX) (Li et al., 2014; Arroussi et al., 2018). Similarly, in bean plants, treatment with *C. vulgaris* culture increased stomata closure frequency and water use efficiency, thereby reducing transpiration and increasing drought tolerance (Li et al., 2014). However, further investigation of exact molecular mechanism and algal determinant for improving abiotic stress tolerance in plant will be required.

**TABLE 2:** Enhancement of abiotic stress tolerance and anti-aging capacity of plants after application of algae.

Objective	Group	Algae species	Plant	Treatment	Product applied	Plant response	References
Abiotic stress tolerance	Cyanobacteria	*Scytonema hofmanni*	Rice	Soil application	Extracellular products	Salt stress tolerance	Rodríguez et al., 2006
	Eukaryotic microalgae	*Chlorella ellipsoida*	Wheat	Soil application	Water soluble extract	Enhanced salt tolerance and antioxidant capacity	Abd El-Baky et al., 2010
		*Chlorella vulgaris*	*Vicia faba* L.	Foliar application	Cell culture	Enhanced reactive oxygen species (ROS) production and more effective stomatal closure and water use efficiency	Li et al., 2014
		*Dunaliella salina*	Tomato	Foliar application	Polysaccharides	Salt stress tolerance	Arroussi et al., 2018
Anti-aging	Eukaryotic microalgae	*C. vulgaris*	Strawberry, lettuce, beet, and kale	Foliar or soil application	Cell culture	Improved shelf-life	Kim et al., 2014
		*Chlorella fusca* and *Chlorella* strains ABC001 and HS2	*Erinus alpinus* L.	Soil application	Cell-free supernatant	Delayed plant senescence	Lee et al., 2020b

### Algae as Biofertilizers

Prokaryotic cyanobacteria have been applied to monocots and dicots as biofertilizers to increase plant growth and crop yield (Figure 2 and Table 3). For example, rice plants treated with *A. variabilis* and *Nostoc* sp. VICCRI via root-drench application showed greater plant height, leaf length, and grain yield than inorganic fertilizer (Singh and Datta, 2007; Innok et al., 2009). Inoculation with *A. laxa* and *Calothrix elenkinii* increased the germination of coriander seeds and promoted root and shoot growth in coriander, cumin, and fennel (Kumar et al., 2013). Soaking of seeds in a solution of *Spirulina platensis* (2 × 10^4^ cells/ml) increased the fresh and dry weight, height, and root length of crop plants, including rocket, Bayam red, and Pak choi, by 1.2–3-fold compared with the untreated control (Wuang et al., 2016). In addition, filtrated supernatant of cyanobacteria *Calothrix* sp., *Hapalosiphon* sp., *Nostoc* sp., and *Westiellopsis* sp., increased coleoptile and radicle length and seed germination in wheat by 2. 7-, 2. 1-, and 1.1-fold, respectively, compared with the sterile water control (Karthikeyan et al., 2009). Interestingly, treatment with multiple species of nitrogen-fixing cyanobacteria has a greater impact on plant growth, probably via synergistic effects on nutrient production (Karthikeyan et al., 2007; Paudel et al., 2012).

**TABLE 3:** Plant growth promotion following algal treatment.

Group	Algae species	Host plant	Treatment	Product applied	References
Prokaryotic cyanobacteria	*Calothrix ghosei, Hapalosiphon intricatus, Nostoc muscorum, Westiellopsis prolifica, Calothrix membranacea*	Wheat	Seed soaking	Filtrated supernatant	Karthikeyan et al., 2009
	*Anabaena laxa and Calothrix elenkinii*	Coriander, cumin, and fennel	Soil application	Cell culture	Kumar et al., 2013
	*Nostoc* sp. VICCRI	Rice	Soil application	Cell culture	Innok et al., 2009
	*Anabaena variabilis*	Rice	Soil application	cell culture	Singh and Datta, 2007

Eukaryotic green algae	*Chlorella vulgaris*	Wheat	Foliar application	Water soluble extract	Shaaban, 2001a
	*C. vulgaris*	Maize	Soil application	Water soluble extract	Shaaban, 2001b
	*C. vulgaris*	Lettuce	Soil application	Dried cell extract	Faheed and Fattah, 2008
	*C. vulgaris, Scenedesmus quadricauda*	Tomato	Hydroponic system	Co-cultivation with plant	Barone et al., 2019
	*C. vulgaris, S. quadricauda*	Sugar beet	Hydroponic system	Dried cell extract	Barone et al., 2018
	*C. vulgaris*	Tomato and cucumber	Seed soaking	Cell culture	Bumandalai and Tserennadmid, 2019
	*Chlorella fusca*	Barely, wheat, lettuce, pepper, melon, cucumber, perilla, onion, radish, and turnip	Soil application	Cell culture	Kim et al., 2012
	*C. fusca*	Spinach	Foliar or soil application	Cell culture	Kim et al., 2018b
	*Chlorella pyrenoidosa*	Soybean	Soil application	Cell culture	Dubey and Dubey, 2010
	*Chlorococcum infusionum*	Tomato	Hydroponic system	Co-cultivation with plant	Zhang et al., 2017
	*Nannochloropsis oculata*	Tomato	Soil application	Dried cell extract	Coppens et al., 2016

Microalgae consortia	*Chlorella, Scenedesmus, Chlorococcum, Chroococcus, Phormidium, Anabaena, Westiellopsis, Fischerella*, and *Spirogyra*	Wheat	Soil application	Cell culture	Renuka et al., 2016
	*C. ghosei, H. intricatus*, and *Nostoc* sp.	Wheat	Soil application		Karthikeyan et al., 2007
	*Nostoc, Anabaena, Westiellopsis, Aulosira*, and *Scytonema*	Rice	Soil application	Cell culture	Paudel et al., 2012

Cyanobacteria–other microbe consortia	Unidentified cyanobacteria and rhizobacteria	Wheat	Soil application	Cell culture	Nain et al., 2010
	*Anabaena oscillarioides* CR3, *Brevundimonas diminuta* PR7, and *Ochrobactrum anthropi* PR10	Rice	Soil application	Cell culture	Rana et al., 2015
	*A. torulosa* and *Trichoderma viride*	Maize	Soil application	Extracted biofilms	Sharma et al., 2020

Similar to cyanobacteria, eukaryotic *Chlorella* spp. increased the growth of *Perilla*, onion, lettuce, Chinese cabbage, radish, turnip, and spinach plants when applied to roots and leaves (Kim et al., 2012, 2018a) (Figure 2 and Table 3). Seed treatment with *C. vulgaris* promoted germination and shoot and root weights in lettuce, tomato, and cucumber (Faheed and Fattah, 2008; Bumandalai and Tserennadmid, 2019). In the field, root-drench application of *Chlorella pyrenoidosa* increased the shoot weight and grain yield of soybean plants by 70 and 53%, respectively, compared with control plants (Dubey and Dubey, 2010). Cell extracts of *C. vulgaris* and dried biomass suspension of *Nannochloropsis oculata* showed plant growth-promoting activity in wheat, maize, tomato, and sugar beet (Shaaban, 2001a, b; Coppens et al., 2016; Barone et al., 2018). Interestingly, recent studies show that co-cultivation of sugar beet and tomato plants with *C. vulgaris*, *Chlorococcum infusionum*, and *Scenedesmus quadricauda* using the hydroponic system simultaneously increases the biomass of both the host plant and eukaryotic algal species (Zhang et al., 2017; Barone et al., 2018, 2019).

### Plant Anti-aging Agents

Treatment with algae or algal solution also affect plant senescence (Figure 2 and Table 2). The ability to prolong plant development and delay the onset of age-related phenotypes is economically important in crop science and critical for fundamental plant research (Lim et al., 2007; Shahri and Tahir, 2014). During initial development of this anti-aging method, treatment with beneficial bacteria such as *Pseudomonas* spp. and *Bacillus* spp. was used to delay plant senescence (Ali et al., 2012; Carlson et al., 2015; Kuan et al., 2016; Naing et al., 2017). Interestingly, spray and irrigation application of *C. vulgaris* culture prolonged the shelf-life of strawberry, lettuce, beet, and kale (Kim et al., 2014). In addition, we reported that root-drench application of the cell-free supernatant of *C. fusca*, *Chlorella* sp. HS2 and *Chlorella* sp. ABC001, delayed shoot and flower senescence by up to 4 weeks in the ornamental flowering plant *Erinus alpinus* (Lee et al., 2020b). Given that other beneficial bacteria modulate ethylene signaling in plants (Ali et al., 2012; Carlson et al., 2015; Kuan et al., 2016; Naing et al., 2017), it is possible that microalgae suppress the ethylene signaling or biosynthesis pathway in plants. The detailed mechanism by which *Chlorella* mediates anti-aging effects in plants is, however, largely unknown.

## Algal Determinants of Plant Health

### Inhibitory Compounds Effective Against Pathogenic Microbes and Insect Pests

**FIGURE 3:**
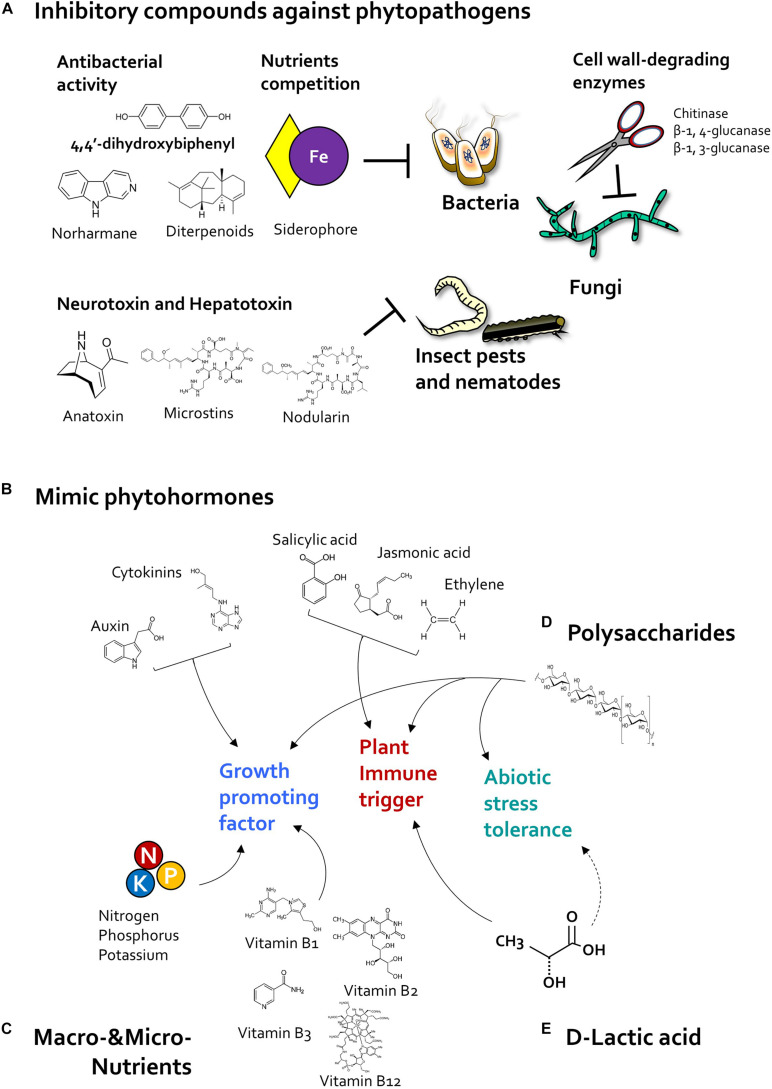
Algal determinants that act as plant protectants and stimulants. **(A)** Inhibitory compounds. Cyanobacteria reduce the population of pathogenic bacteria, fungi, and insect pests by producing antibiotic and pesticidal compounds. Cyanobacteria-derived 4,4′-dihydroxybiphenyl, norharmane, and diterpenoids exhibit antibacterial activity, and microalgal siderophores inhibit bacterial growth through iron (Fe) competition. In addition, cyanobacterial cell wall-degrading enzymes such as chitosanase, β-1,4-glucanase, and β-1,3-glucanase reduce fungal infection. Cyanotoxins such as anatoxin, microcystin, and nodularin can protect the host plant against insect pests. **(B)** Phytohormones. Microalgae-derived phytohormone-mimicking compounds modulate plant growth, immunity, and abiotic stress tolerance. Plant growth regulators such as auxin and cytokinin increase plant growth and development as well as crop yield. Algae species also produce jasmonic acid (JA), salicylic acid (SA), and ethylene (ET), which act as major defense-related hormones in land plants. In addition, microalgae also produce abscisic acid (ABA), a central regulator of abiotic stress tolerance. **(C)** Nutrition. Nitrogen-fixing cyanobacteria promote plant growth by supplying macronutrients such as nitrogen, phosphorus, and potassium. Additionally, microalgae-derived vitamins, including vitamins B1, B2, B3, and B12, elicit plant immune response against phytopathogens. **(D)** Polysaccharides. Polysaccharides extracted from cyanobacteria and eukaryotic microalgae increase immunity and abiotic stress tolerance of the host plant. **(E)** D-lactic acid. Exogenous application of D-lactic acid produced by *Chlorella* elicits plant immunity via activation of D-lactate metabolism and production of mitochondrial reactive oxygen species (ROS). Algal D-lactic acid might also enhance abiotic stress tolerance in host plant by regulating ROS production.

Like classic bacterial biocontrol agents, beneficial algae produce antimicrobial compounds that suppress bacterial and fungal plant pathogen (Figure 3). For example, 4,4′-dihydroxybiphenyl, norharmane prokaryotic algae *Nodularia* spp. *and Nostoc* spp. and *Nostoc insulare* produces 4,4′-dihydroxybiphenyl, norharmane, and diterpenoids, which exhibit antibacterial activity against *Escherichia coli* and *Pseudomonas aeruginosa, Bacillus subtilis, B. cereus*, *Staphylococcus epidermdis* (Jaki et al., 2000; Volk and Furkert, 2006). In addition, cyanobacteria *Anabaena* spp., *Chlorella* spp., and *Scenedesmus* spp., produced siderophore as micronutrient ferric and copper ion chelators (McKnight and Morel, 1980; Goldman et al., 1983; Benderliev, 1999; Benderliev et al., 2003). Siderophores produced by microbes, especially such as *Pseudomonas* spp., were known as antimicrobial compounds and biological control agents in plants via chelating ferric iron, which can compete with bacterial pathogens for iron ions (Kloepper et al., 1980; Duijff et al., 1993; Lemanceau and Alabouvette, 1993). On the other hand, prokaryotic algae cyanobacteria can produce fungal cell wall-degrading enzymes including chitosanase, β-1,4-glucanase, β-1,3-glucanase, and benzoic acid, which can suppress growth of *Fusarium* sp., *Penicillium* sp., and *Candida* sp. (López et al., 2002; Chaudhary et al., 2012; Natarajan et al., 2012; Prasanna et al., 2013, 2016). Thus, further identification of microalgal antimicrobial compounds, and their biological control activity, is needed.

In addition to antimicrobial substances, cyanobacteria also produce pesticidal and nematocidal secondary metabolites, referred to as cyanotoxins (Hamouda and El-Ansary, 2017) (Figure 3). Cyanotoxins function as neurotoxins and hepatotoxins in animals (Sathiyamoorthy and Shanmugasundaram, 1996; Holajjer et al., 2013). The neurotransmitter-mimicking cyanotoxin, anatoxin-a, binds to eukaryotic nematode receptors and triggers continuous muscle contraction, causing muscle fatigue, and immobility (Carmichael, 1994; Dow and Swoboda, 2000; Mankiewicz et al., 2003). Moreover, cyanobacteria *Microcystis* spp. produce hepatotoxins including microcystins and nodularin (Holajjer et al., 2013), which inhibit the host metabolic system; for example, nodularin produced by *Nodularia spumigena* inhibit protein phosphatase activity in animal cells (Ohta et al., 1994). Additionally, cyanobacteria also produce peptide toxins that act as repellents (Sathiyamoorthy and Shanmugasundaram, 1996); for example, *Anabaena* and *Scytonema* species produce a low molecular weight (<12 kDa) peptide toxin (Konst et al., 1965; Sathiyamoorthy and Shanmugasundaram, 1996). Interestingly, *Scytonema*-derived peptide toxin acts as a repellent due to its strong smell, and reduces the population size of chewing insects *Helicoverpa armigera* and *Stylepta derogate* on cotton leaves (Sathiyamoorthy and Shanmugasundaram, 1996). Collectively, these reports suggest that algal substances can inhibit phytopathogenic bacteria, fungi, pests, and nematodes directly. However, most of these algal compounds exhibit antagonistic activity against phytopathogens only *in vitro*. Thus, it is important to verify the activity of purified algal compounds *in planta*.

### Plant Hormone-Mimicking Compounds

The plant growth-promoting microalgae, including prokaryotic cyanobacteria and eukaryotic microalgae, produce phytohormones such as auxin and cytokinin, which affect plant growth and development (Werner et al., 2001; Benjamins and Scheres, 2008).

Auxin regulates plant developmental processes including gametogenesis, embryogenesis, seedling growth, vascular patterning, and flower development (Hamann et al., 2002; Dimitrov and Zucker, 2006; Pagnussat et al., 2009). Auxins, including indole-3-acetic acid (IAA), indole-3-butyric acid (IBA), indole-3-propionic acid (IPA), and 3-methylindole, have been detected in diverse microalgae species (Misra and Kaushik, 1989; Mazur et al., 2001; Stirk et al., 2002, 2013; Karthikeyan et al., 2009; Hashtroudi et al., 2013). Interestingly, algal auxin seems to positively regulate plant–algae interactions (Figure 3). IAA produced by *Nostoc* species promotes plant growth in wheat and rice; a *Nostoc* mutant lacking the IAA biosynthesis gene, which encodes indole pyruvate decarboxylase, failed to promote plant growth (Hussain et al., 2013, 2015). In addition to plant growth promotion, algal auxin is also tightly linked with the ability of microalgae to colonize host roots (Ahmed et al., 2010; Hussain et al., 2013, 2015). Auxin production in *Leptolyngbya* sp. MMG-1, *Chroococcidiopsis* sp. MMG-5, and *Synechocystis* sp. MMG-8, was increased during their colonization of plant roots (Ahmed et al., 2010). Strikingly, the lack of indole pyruvate decarboxylase significantly reduced colonization of rice and wheat roots by *Nostoc* species (Hussain et al., 2013, 2015). Collectively, algal auxin might act as a putative signaling molecule that mediates plant–microalgae interactions.

Cytokinin promote division and differentiation of plant cells, especially in apical and axillary meristems, and development of gynoecium, and female gametophyte (Marsch-Martínez et al., 2012; Cheng et al., 2013; Schaller et al., 2014). Cytokinin compounds, including *trans*-zeatin, *cis*-zeatin, zeatin riboside, dihydrozeatin riboside, topolin, and zeatin-*o*-glucoside, were produced by many microalgae species (Stirk et al., 2002, 2013; Tsavkelova et al., 2006; Hussain et al., 2010; Hussain and Hasnain, 2011). Similar with auxin, algal cytokinin also positively regulated plant growth promotion and root colonization (Figure 3). Knockout mutant of the cytokinin biosynthesis gene, which encodes isopentenyl transferase, in the plant growth-promoting cyanobacterium *Nostoc* AHM-12 failed to increase plant growth, and significantly reduced root colonization in rice and wheat (Hussain et al., 2013). Thus, in addition to auxin, understanding the molecular basis of how algal-derived cytokinin influence plant–algae interactions will be an interesting topic for future research.

In addition to growth-promoting phytohormone, defense-related hormones such as jasmonic acid (JA), salicylic acid (SA), and ethylene (ET) are produced by algae species (Rodgers et al., 1979; Kreslavsky et al., 1997; Tsavkelova et al., 2006; Natarajan et al., 2012). Plant immunity can be activated systemically by PGPR, depending on JA, SA, and ET signaling (Pieterse et al., 1998, 2014; De Meyer et al., 1999; Kloepper et al., 2004; van Loon et al., 2006) (Figure 3). In addition, algae treatment can also induce activation of defense hormone signaling in host plant. Foliar application of the supernatant of *C. fusca* activates SA and JA signaling upon pathogen inoculation in *Arabidopsis* (Lee et al., 2020a). Similarly, treatment with liquid extracts of eukaryotic *Tetraselmis* sp., *D. salina*, *N. gaditana*, *Aphanothece* sp., and *A. maxima* induce the accumulation of the JA precursor, linolenic acid, in tomato (Mutale-joan et al., 2020). Thus, plant immunity triggered by algae is tightly involved in activation of defense-related hormonal signaling.

### Polysaccharides

Algae produce diverse polysaccharides as cell wall components. Given their medical and cosmetic applications, algal polysaccharides are recognized as important substances (Figure 3). To utilize polysaccharides to improve plant health, studies have been conducted to gain molecular insight into the role of agal polysaccharides in plant protection (Arroussi et al., 2018; Farid et al., 2019). Bacterial and fungal polysaccharides such as lipopolysaccharides (LPSs) and EPSs are plant immune elicitors (Erbs and Newman, 2003; Park et al., 2008). Sulfated EPSs produced by *D. salina* increase salt stress tolerance, expression of genes encoding antioxidant enzymes (CAT, POD, and SOD), and accumulation of JA precursor in tomato (Arroussi et al., 2018). Crude polysaccharides extracted from *Chlorella vulgaris*, *Chlorella sorokiniana*, and *Chlamydomonas reinhardtii* increase expression of *PR* genes and genes encoding antioxidant enzymes such as β-1,3-glucanase, APX, and POD in tomato plants (Farid et al., 2019).

In addition to immune activation, algal polysaccharides can also improve the growth and abiotic stress tolerance of host plants. The application of algal polysaccharides extracted from cyanobacterium *S. platensis* and *A. platensis*, and eukaryotic *D. salina* and *Porphorydium* sp. promoted shoot and root growth in in tomato and pepper (Elarroussia et al., 2016; Rachidi et al., 2020). Moreover, spray treatment of polysaccharides extracted from *D. salina* increased the shoot dry weight, and root dry weight of tomato plants by 1.8- and 5. 5-, respectively, under high salt stress compared with untreated plants (Arroussi et al., 2018), implying that algal polysaccharides enhance salt tolerance. Compared with microalgae polysaccharides, macroalgal polysaccharides such as carrageenans and beta-glucans (laminarin, ulvan, and fucan) mainly function as biostimulants and bioprotectants (Mercier et al., 2001; Sangha et al., 2010, 2015; Vera et al., 2012; Ghannam et al., 2013; Shukla et al., 2016; Pettongkhao et al., 2019; Zou et al., 2019). However, the structure of microalgal polysaccharides is largely unknown. Thus, to elucidate the mode of action of microalgal polysaccharides in plants, it is important to identify the main determinant(s) in crude polysaccharide algal extracts.

### D-lactic Acid

D-lactic acid is a major compound produced by *Chlorella* species (Gruber et al., 1974; Lee et al., 2020a). Recently, D-lactic acid in the supernatant of *C. fusca* was identified as a determinant of plant immunity against *Pseudomonas syringae* pv. tomato DC3000 in *Arabidopsis* (Lee et al., 2020a) (Figure 3). Especially, foliar application of D-lactic acid primed production of ROS after flagellin 22 (flg22) treatment in *Arabidopsis* (Lee et al., 2020a). Primed ROS production by D-lactic acid might be correlated with D-lactate oxidation and mitochondrial ROS (mtROS) production. D-lactic acid is metabolized by the D-lactate dehydrogenase (D-LDH), which localizes to the intermembrane space of mitochondria (Atlante et al., 2005; Welchen et al., 2016). Activation of D-LDH correlates strongly with activation of mitochondrial antioxidant enzyme (Husic and Tolbert, 1987). In *Arabidopsis*, exogenous application of D-lactic acid increases expression of *D-LDH*, *cytochrome c oxidase subunit 2* (*COX2*), and *alternative oxidase 1* (*AOX1*) in flg22-treated *Arabidopsis* (Lee et al., 2020a). These mitochondrial antioxidant enzymes might be activated to catalyze mtROS produced by D-lactic acid. Thus, microalgal-derived D-lactic acid enhances plant innate immunity and production of mtROS in plant.

The activation of D-LDH by D-lactic acid can also affect abiotic stress tolerance via methylglyoxal (MG) detoxification (Figure 3). MG is a cytotoxic compound generated as a byproduct of glycolysis, which accumulates under abiotic stress conditions (Maurino and Engqvist, 2015). To detoxify the accumulated MG, plants activate the expression of *D-LDH*, which encodes the last enzyme in the MG detoxification pathway (Maurino and Engqvist, 2015). Recently, studies showed that D-LDH-mediated MG detoxification correlates with abiotic stress tolerance in yeast, sorghum, and rice (An et al., 2017; Jain et al., 2018, 2020; Bhowal et al., 2020). In sorghum, the expression of *D-LDH1–4* genes was activated under heat, cold, salt, and drought stress conditions (Bhowal et al., 2020). In rice, *D-LDH* RNA interference (RNAi) plants were more sensitive to salt stress (200 mM NaCl) than wild-type plants (An et al., 2017). However, overexpression of *D-LDH2* conferred tolerance to multiple abiotic stresses, including salt stress, oxidative stress, osmotic stress, and heat stress in rice plants (Jain et al., 2020). Thus, microalgae-derived D-lactic acid might alleviate abiotic stress tolerance in plants via D-LDH-mediated MG detoxification.

### Plant Macro- and Micronutrients

Algae have been utilized as a source of macro- and micronutrients for plants (Figure 3). Microalgae cyanobacteria possess specialized cells called heterocysts, which can fix atmospheric nitrogen (Singh and Bisoyi, 1989; Gantar et al., 1993; Karthikeyan et al., 2007; Babu et al., 2015). Thus, inoculation of soil with nitrogen-fixing cyanobacteria enhanced plant growth by increasing the availability of nitrogen, carbon, and vitamins (Tripathi et al., 2008; Prasanna et al., 2009; Renuka et al., 2016). In addition, application of microalgae consortium comprising *Chlorella*, *Scenedesmus*, *Chlorococcum*, *Chroococcus*, *Phormidium*, *Anabaena*, *Westiellopsis*, *Nostoc*, *Aulosira*, and *Scytonema* to soil enhanced the content of available nitrogen, phosphorus, and potassium (Paudel et al., 2012; Renuka et al., 2016).

Algae also secrete vitamins, which promote plant growth and plant immunity (Havaux et al., 2009; Goyer, 2010) (Figure 3). Previously studies show that bacteria-derived vitamins B1, B2, and K3, act as elicitors of plant immunity against pathogenic fungi, bacteria, and viruses, and that biotin, thiamine, cobalamin, pantothenic acid, and niacin produced by bacteria enhance plant growth (Strzelczyk et al., 1991; Ahn et al., 2005; Taheri and Hofte, 2007; Liu et al., 2010; Taheri and Tarighi, 2010; Song et al., 2013). Cyanobacteria such as *Spirulina*, *Anabaena*, *Microcystis*, *Nostoc*, *Phormidium*, *Oscillatoria*, *Chroococcus*, and eukaryotic algae such as *Euglrena*, also produce thiamine (vitamin B1), riboflavin (vitamin B2), folic acid, ascorbic acid, nicotinic acid (vitamin B3), cyanocobalamin (vitamin B12), and vitamin E (Robbins et al., 1951; Koptera, 1970; Aaronson et al., 1977; Shah and Vaidya, 1977; Gupta et al., 2013). In addition, the extract of *N. muscorum* and *Hapalosiphon* containing vitamin B-complex (including cyanocobalamin, niacin, pantothenic acid, and folic acid) increases coleoptile length and leaf length in rice (Misra and Kaushik, 1989). Since land plants lack vitamin B12, their growth is supported by beneficial microbes containing vitamin B12 (Watanabe and Bito, 2018). Similarly, as beneficial microbes, microalgal species can also alleviate vitamin B deficiency in host plants. Further investigation of the effects of algae-derived macro- and micronutrients in plants is needed.

## Interaction Between Microalgae and Other Microbes in the Plant Microbiome

Algae benefit plants through several mechanisms. In order to consider microalgae as part of the plant microbiome, it is necessary to understand the interactions between microalgae and other plant microbiota. Interestingly, previous reports showed the synergism between algae and bacteria during co-inoculation of plants. A mixture of cyanobacteria and plant-associated eubacteria or fungi additively or synergistically improves the growth and health of diverse crop plants (Tables 1, 3) (Nain et al., 2010; Dukare et al., 2011; Rana et al., 2015; Sharma et al., 2020). Soil inoculation with a mixture containing the cyanobacterium *Anabaena oscillarioides* and plant growth-promoting bacteria *Brevundimonas diminuta* and *Ochrobactrum anthropi* improved rice yield by 1.2-fold compared with the control (Rana et al., 2015). Treatment with a biofilm comprising *A. torulosa* and the plant growth-promoting fungus *Trichoderma viride* increased the seed germination rate and radicle length in maize (Sharma et al., 2020). In addition, the combined application of *Anabaena* spp. and *B. subtilis* reduced the severity of fungal disease caused by *Fusarium*, *Pythium*, and *Rhizoctonia* by twofold compared with the control (Dukare et al., 2011).

The interaction between microalgae and other microorganisms might be governed by interspecific exchange of metabolites (Gonzalez and Bashan, 2000; Kazamia et al., 2012; Kim et al., 2014). Plant-associated rhizobacteria or fungi support the growth and root colonization of microalgae species by providing secondary metabolites such as vitamin B12, siderophores, volatile compounds, *N*-acylhomoserine lactone, and EPSs (Gobler et al., 2007; Choix et al., 2012; Kazamia et al., 2012; Santos and Reis, 2014; Amavizca et al., 2017; Cho et al., 2019; Sharma et al., 2020). In turn, microalgae provide photosynthates, including fixed carbon, as nutrient sources for soil-borne microbes (Gobler et al., 2007; Kazamia et al., 2012). Taken together, these studies imply that exogenous microalgae can interact with other soil–borne microbes in plant microbiome, as do traditional plant-associated bacteria and fungi.

## Rhizosphere Microbiome Engineering With Algae

Modification of the rhizosphere using microalgae, including cyanobacteria and eukaryotic microalgae, will potentially allow us to engineer and change the structure and effectiveness of the rhizosphere microbiome, thereby improving plant health. Previously, the effect of soil algae diversity on plants was investigated by application of a commercial proprietary suspension of microalgae called GOgreen^®^ (Hastings et al., 2014). Four algal groups, including green algae (Chlorophyta), blue–green algae (Cyanophyta), yellow–green algae (Xanthophyta), and diatoms (Bacillariophyta), are mainly found in soil (Paul and Clark, 1989). The application of GOgreen^®^ to maize roots under field conditions increased the number and diversity of diatoms and reduced the soil pH with a pH higher than 7. Since the connection between species diversity and their influence on ecological function is unclear, the authors measured two indicators of soil quality: organic matter content (OM) and cation exchange capacity (CEC). The values of OM and CEC were improved significantly by algae treatment (Hastings et al., 2014). In addition, inoculation of rice plants with the cyanobacterium *Calothrix elenkenii* increased the bacterial population diversity in the microbiome by 10-fold (Priya et al., 2015). Fatty acid methyl ester analysis and 16S rRNA sequencing data indicated that Bacillaceae was the most abundant bacterial group induced by cyanobacteria inoculation. Moreover, *C. elenkenii* inoculation increased the shoot length, root length, fresh weight, and dry weight of plants as well as enhanced the level of plant hormones (IAA and ABA), chlorophyll, and antioxidant enzymes (POD, polyphenol oxidase [PPO], and PAL). However, direct evidence based on experiments using the gnotobiotic system was not provided. In the line of this study, more direct approaches were also attempted. Next-generation sequencing of 16S rRNA amplicons was conducted to determine the effect of *C. vulgaris* application on bean root microbiota (Kublanovskaya et al., 2019). Interestingly, no significant changes were detected in bacterial diversity in the bean rhizosphere upon the application of *C. vulgaris*. Algae-mediated microbiome engineering for promoting plant health is in its infancy. Fine-tuning microbiome engineering for keystone taxa that affect plant growth and health is necessary, and algae and their products can be utilized for this purpose.

A synthetic microbiome comprising algae and bacteria represents a promising tool for the sustainable development of soil fertility, water preservation, and plant growth, especially under stress conditions (Nain et al., 2010; Rana et al., 2015; Perea et al., 2018). A consortium of eukaryotic microalgae, cyanobacteria, and bacteria will provide organic carbon for plant growth (Belnap, 2003; Bashan and de-Bashan, 2010), fix atmospheric nitrogen (Issa et al., 2001; Pointing and Belnap, 2012), and promote seedling survival (Godínez-Alvarez et al., 2012). Detailed investigation of the algae–bacteria network and their effect on the plant microbiome is required to maximize plant growth and protect plants against pathogens (Krug et al., 2020).

## Potential Applications of Algae

The beneficial effects of algae on plants and agriculture have been described above. Large-scale production of algae has been optimized for improving human health; however, the application of algae for large-scale crop cultivation has not been elucidated. We summarized the determinants of algae that augment plant growth and immunity, and classified these determinants as secreted products and the cell itself (Figure 2). The inoculation of plants with cell wall components such as glucans, increased plant growth and activated plant defense responses (Mercier et al., 2001; Sangha et al., 2010, 2015; Vera et al., 2012; Ghannam et al., 2013; Shukla et al., 2016; Pettongkhao et al., 2019; Zou et al., 2019, Figure 3). The products secreted by algae can be harvested in large amounts when algae are grown in liquid media. D-lactic acid was recently identified as an algal determinant that elicits plant immune response against bacterial pathogens (Lee et al., 2020a, Figure 3). Additionally, plant defense hormone-mimicking compounds, such as JA, benzoic acid and ET, were also detected in algae culture (Rodgers et al., 1979; Kreslavsky et al., 1997; Tsavkelova et al., 2006; Natarajan et al., 2012, Figure 3). These defense hormones strongly activate plant defense when supplied exogenously. Cell and cell envelope components of algae can be used for limited applications in the greenhouse and field to reduce the high production cost, although these products demonstrate high efficacy (Choleva et al., 2005, 2007; Dubey and Dubey, 2010; Bileva, 2013; Coppens et al., 2016). Products secreted in the liquid culture of algae also show a great potential for application in the field (Shaaban, 2001a, b; Barone et al., 2018; Mutale-joan et al., 2020). Generally, large-scale production of algae, mostly by heterotrophic cultivation, is performed to harvest algal cells (Lee et al., 2020a). The cell-free components are considered waste products that need to be detoxified. If the cell-free extracts can be reused for plants, their potential applications will increase greatly.

However, several issues must be addressed prior to application of algae on crop plants. First, the potential harmful effects of cell-free extracts of algae should be evaluated and eliminated. In many cases, algae produce toxic compounds during cultivation. For instance, at high concentrations, 2,4-D (auxin) acts as a herbicide (Marth and Mitchell, 1944). Thus, quality control of the liquid culture of algae is critical. Second, production of algal determinants should be optimized for large-scale production. Third, formulation of cell-free extracts should be carefully considered. The cell-free extract could simply be applied by drench application or by using the drip-irrigation system. However, the delivery of a large volume of extract is problematic. Therefore, the extract should be vaporized and purified using chemical and physical procedures, and the final product showing high effectiveness should be used for agricultural applications. Furthermore, granulation of determinants is similar to that of other agricultural products such as fertilizers and agrochemicals. Finally, the specific procedure how to isolate effective algae in plant health improvement also must be developed in near future.

Previously, algae were not considered as a member of the beneficial plant microbiome. However, with recent progress in metagenome analysis, algae are now recognized as important members of the plant microbiome. While microbes such as bacteria and fungi have been used to improve plant fitness, new data indicate that algae also promote plant growth and act as biological control agents against pathogens by directly inhibiting pathogen growth and activating plant immune responses. Thus, algae represent a new bioactive material that can be utilized as biofertilizers and plant protectants, which implies that algae should be classified as a member of the beneficial plant microbiome.

## Author Contributions

C-MR designed the review. S-ML created the figures and tables. C-MR and S-ML wrote the manuscript. Both authors contributed to the article and approved the submitted version.

## Conflict of Interest

The authors declare that the research was conducted in the absence of any commercial or financial relationships that could be construed as a potential conflict of interest.
